# Use of Electropolishing in Orthodontic Appliances: An Option to Reduce the Risk of Metal Hypersensitivity

**DOI:** 10.3390/dj12070193

**Published:** 2024-06-24

**Authors:** Edith Lara-Carrillo, Ulises Velázquez-Enríquez, Brenda Andrea Ramírez-García, María Fernanda Lara-Fonseca, Raúl A. Morales-Luckie, Ana Miriam Santillán-Reyes, Victor Hugo Toral-Rizo, Elias Nahúm Salmerón-Valdés, Adriana Alejandra Morales-Valenzuela

**Affiliations:** 1 Center for Research and Advanced Studies in Dentistry, School of Dentistry, Autonomous University of Mexico State, Toluca de Lerdo 50130, Mexico; vhtoralr@uaemex.mx; 2 School of Dentistry, Autonomous University of Mexico State, Toluca de Lerdo 50130, Mexico; bramirezg833@alumno.uaemex.mx (B.A.R.-G.); mlaraf449@alumno.uaemex.mx (M.F.L.-F.); amsantillanr@uaemex.mx (A.M.S.-R.); ensalmeronv@uaemex.mx (E.N.S.-V.); aamoralesv@uaemex.mx (A.A.M.-V.); 3 Department of Nanomaterials, Sustainable Chemistry Research Center, National Autonomous University of Mexico/Autonomous University of State of Mexico, Toluca de Lerdo 50200, Mexico; rmoralesl@uaemex.mx

**Keywords:** nickel, orthodontics, hypersensitivity, electropolishing, metals

## Abstract

Electropolishing is a common treatment in the industry; however, how it behaves in the mouth and what benefits it can bring over metal dental attachments have not yet been established. Thus, the aim of this study was to determine the levels of corrosion, the released metal ions, and the changes in structural composition in metallic orthodontic appliances following electropolishing treatment. This study included 56 orthodontic brackets and 28 archwires. The samples were subjected to a pH cycle to simulate an oral environment. Using UV–Vis spectrophotometry, the release of metallic particles was evaluated, and using scanning electron microscopy, the structural and composition changes were evaluated. Groups were compared using Student’s *t*-tests with a value of *p* ≤ 0.05. The cyclical pH solutions showed variations between groups and days (1, 3, 5, 7 and 15), reaching the highest acidification in the self-ligating brackets; the absorbance between solutions did not differ significantly. As seen from the SEM results, the experimental group showed minor irregularities compared with the control groups. The experimental brackets decreased in iron and increased in chromium after electropolishing, while for the NiTi archwires, they decreased in nickel. Therefore, electropolishing treatments in metallic orthodontic attachments improve their surface structure and corrosion resistance could reduce the risk of metal hypersensitivity, mainly from nickel.

## 1. Introduction

Previous research showed that the surface of orthodontic appliances is affected by several factors such as saliva flow, chewing, brushing, friction between the brackets and wires, acidic drinks, mouthwash, toothpaste, biofilm formation, and oral pH fluctuations (from 5.2 to 7.89), among others [1,2].

Metallic orthodontic appliances are composed principally of stainless steel containing 8 to 12% nickel, 17 to 22% chromium, and various proportions of manganese, copper, titanium, and iron. Stainless steel is also used with a wide range of applications in fixed and removable appliances [3]. Meanwhile, nickel–titanium wires have the unique properties of retaining shape memory and being superelastic [4].

Corrosion is the graded degradation of materials following an electrochemical attack and are concerning, particularly when orthodontic appliances are placed in a hostile electrolytic environment such as the human mouth [5,6]. Even though a protective oxide film exists on metal surfaces, metal ions can still be released in the oral cavity through corrosion processes and may have adverse biological effects, depending on the ion type and concentration [7].

Animal research has shown that a relatively high concentration of nickel is needed to produce toxic effects, but even at low concentrations, this metal can produce an allergic reaction [4]. The literature indicates that approximately 10% of the population is sensitive to nickel, which is more commonly observed in females (1.6% of females and 0.2% of males) [8].

Industrial procedures used to reduce the corrosion of metals such as electrochemical polishing or electropolishing could thus be an effective method of surface treatment [9]. Some advantages of electropolishing are listed below:

- Gives a better aesthetic appearance;
- Allows anti-corrosion properties to be obtained due to the formation of a passive oxide layer with chromium oxide (Cr_2_O_3_), nickel oxide (NiO), molybdenum oxide (Mo_2_O_3_), and iron oxide (Fe_2_O_3_);
- Facilitates cleaning and washing (improves bacteria and dirt removal);
- Reduces microstresses in the superficial layer by processing and restoring uniform micro-hardness of the native material;
- Polishes surfaces and spots inaccessible to mechanical polishing [10].

Electropolishing treatments on metal surfaces are a very common treatment in the industry, but in dentistry, it is only beginning to be used; furthermore, how it behaves in the mouth and what benefits, not only structural but also physiological, it brings over metal attachments have yet to be established.

Additionally, orthodontics is a treatment that lasts approximately 2–3 years in a patient’s mouth, which has been seen to cause corrosion, the release of ion metals, even some alterations at the cellular level [11,12], and, in some patients, hypersensitivity to some metals, mainly nickel. Thus, having a method that can reduce these risk factors is important, and electropolishing has been suggested as an option.

Therefore, this study aimed to determine the levels of corrosion, released metal ions, and changes in structural composition in metallic orthodontic appliances following electropolishing treatments as an option to reduce metal hypersensitivity.

## 2. Materials and Methods

### 2.1. Sample Selection

In this longitudinal study, 56 brackets were divided into experimental groups, with 28 metal brackets (14 self-ligating and 14 Roth prescriptions), and control groups, with 28 metal brackets (14 self-ligating and 14 Roth prescriptions).

Then, 28 archwires were selected and cut in half, obtaining 56 archwire halves, which were subsequently divided into two groups: controls, with 28 halves of NiTi and stainless steel archwires, and experimental groups, also with 28 halves of NiTi and stainless steel archwires, each with two different calibers.

The experimental groups received electropolishing treatments, and the control groups remained as obtained directly from the manufacturer.

Bracket groups

Control groups:

- GA: 10 brackets, upper right hemiarch and lower right hemiarch (3M^TM^ Unitek^TM^ Victory SL self-ligating slot 0.022”, Monrovia, CA, USA);
- GB: 10 brackets, upper right hemiarch and lower right hemiarch (TP Orthodontics, Nu-Edge Roth slot 0.018”, La Porte, IN, USA).

Experimental groups:

- GC: 10 brackets, upper left hemiarch and lower left hemiarch (3M^TM^ Unitek^TM^ Victory SL self-ligating slot 0.022”, Monrovia, CA, USA);
- GD: 10 brackets, upper left hemiarch and lower left hemiarch (TP Orthodontics, Nu-Edge Roth slot 0.018”, La Porte, IN, USA).

Archwire groups

Control groups:

- GE: 7 NiTi wire halves, 0.018” (TP Orthodontics, La Porte, IN, USA);
- GF: 7 NiTi wire halves, 0.016 × 0.022” (TP Orthodontics, La Porte, IN, USA);
- GG: 7 stainless steel wire halves, 0.016” (TP Orthodontics, La Porte, IN, USA);
- GH: 7 stainless steel wire halves, 0.017 × 0.025” (TP Orthodontics, La Porte, IN, USA).

Experimental groups:

- GI: 7 NiTi wire halves, 0.018” (TP Orthodontics, La Porte, IN, USA);
- GJ: 7 NiTi wire halves, 0.016 × 0.022” (TP Orthodontics, La Porte, IN, USA);
- GK: 7 stainless steel wire halves, 0.016” (TP Orthodontics, La Porte, IN, USA);
- GL: 7 stainless steel wire halves, 0.017 × 0.025” (TP Orthodontics, La Porte, IN, USA).

### 2.2. Preparation of the Experimental Group (Electropolishing)

For electropolishing, one litre of electrolyte solution was placed in an electropolishing tub (Molident, Mexico City, Mexico), and the brackets and archwires were submerged and attached to the stem for 10 s at 12 V, with a temperature of 40 °C and a current density of 5 A/dm^2^.

### 2.3. pH Cycle

The samples were subjected to a pH cycle to simulate an oral environment: the processes of chewing and resting in the oral cavity were simulated through shifts between demineralizing and remineralizing solutions. The demineralization of teeth occurs when the acidic by-product of plaque wears away at the enamel of the teeth, while during the remineralization, the body takes calcium and phosphate minerals from saliva and deposits them in dental enamel, avoiding the consequent formation of cavities. The remineralizing solutions were composed of 1.5 mM CaCl_2_, 0.9 mM Na_2_PO_4_, and 0.15 mM KCl, and the demineralizing solutions were composed of 2.2 mM CaCl_2_, 2.2 mM NaH_2_PO_4_, 0.05 nM acetic acid, 1 nM of KOH; both solutions were prepared according to the method of Prado et al. [13]. The samples were kept at 37 °C for 15 days under the pH cycle, with 3 h of demineralization and 21 h of remineralization.

### 2.4. Spectrophotometry and pH

The release of metal particles from the brackets and archwires in the remineralizing and demineralizing solutions was quantified using a UV–Vis Spectrophotometer (PerkinElmer, Inc., Lamda 25, Waltham, MA, USA) at a wavelength of 190 nm−370 nm.

**Figure 1:**
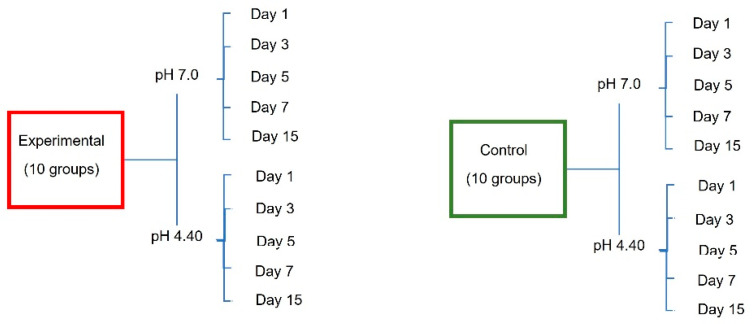
Distribution of the study groups.

After the end of the pH cycle, the particles present in each solution tube on days 1, 3, 5, 7 and 15 were measured, resulting in 3 solution samples from each group, a total of 600 analyzed samples (Figure 1), and a change in the acidity/alkalinity of these solutions.

### 2.5. SEM Characterization and EDS Analysis

Through scanning electron microscopy (SEM), at the end of the pH cycle, the structural changes and the atomic percentages of the metals were evaluated in both cases (with and without electropolishing) using a scanning electron microscope (model JSM-6510LV, JEOL, Ciudad de México, México) at a magnification of 800×–2000× and 20 kv.

### 2.6. Statistical Analysis

The results were analysed with SPSS version 23, and the data were checked for normal distribution using a Shapiro–Wilk test; furthermore, Student’s *t*-tests between the groups were employed, with a 95% confidence interval (−0.2606–0.1444) and a significance level of *p* ≤ 0.05.

## 3. Results

### 3.1. pH of Remineralizing and Demineralizing Solutions

**Figure 2:**
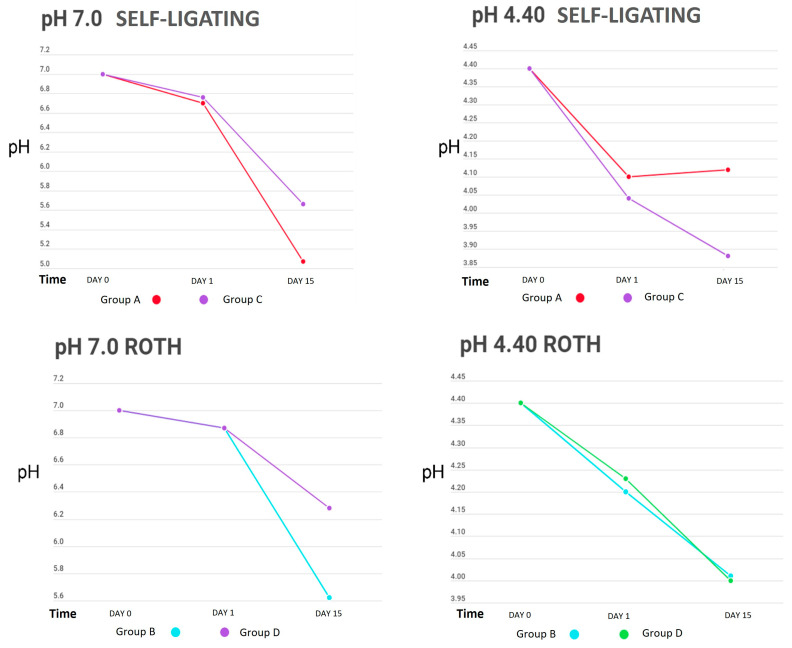
pH measurements of remineralizing (pH = 7.0) and demineralizing (pH = 4.40) solutions in bracket groups.

**Figure 3:**
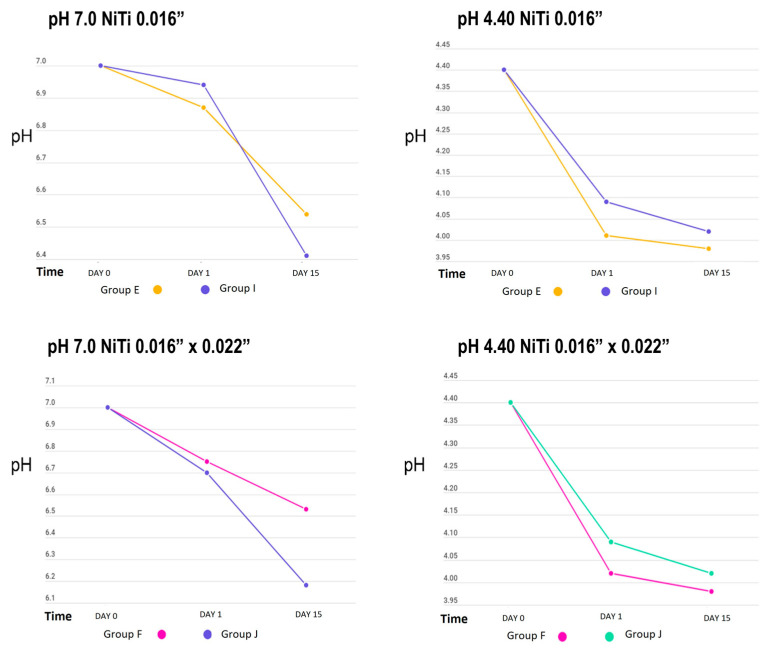
pH measurement of remineralizing (pH = 7.0) and demineralizing (pH = 4.40) solutions in NiTi archwire groups.

**Figure 4:**
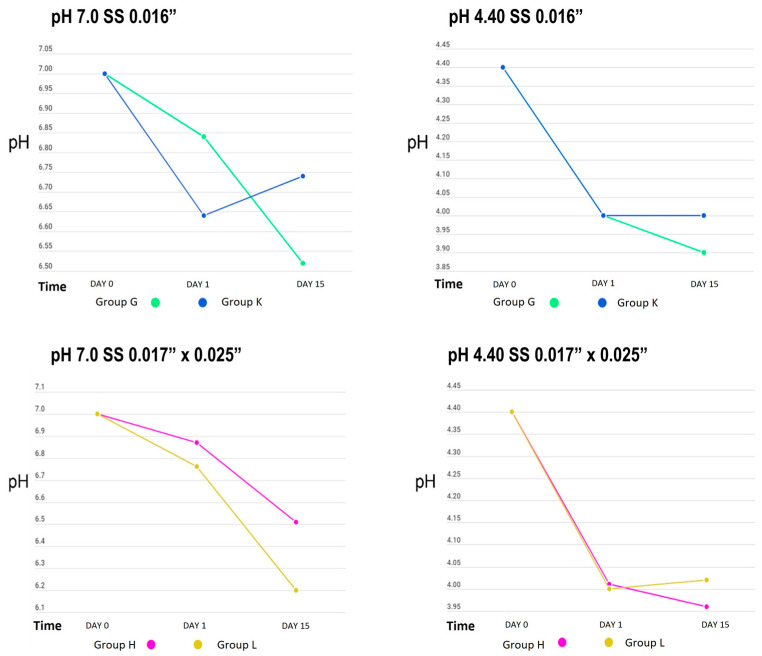
pH measurement of remineralizing (pH = 7.0) and demineralizing (pH = 4.40) solutions in SS archwire groups.

Table 1 shows the behaviour of the cyclical pH solutions over time in the different groups. The remineralizing solution, with a pH of 7.0, presented variations; the self-ligating brackets (GA) showed a decrease in pH up to 5.07 vs. the experimental group (GC), at a pH of 5.66, on day 15. The group with greater stability was GI, with a pH of 6.94, vs. the control group (GE), with a pH of 6.87, on the first day of testing (Figure 2, Figure 3 and Figure 4).

The initial demineralizing solution had a pH of 4.40, which decreased to 3.88 in GC and to 4.04 in GA, for both self-ligating brackets on day 15. The least amount of change was in GD, to 4.23, and GB, to 4.20 (Roth-type brackets), on day 1. However, a basification of this solution was also observed, increasing up to 5.25 in GK (0.016” SS arches) and 5.20 in GG.

When we analysed the average behaviour of the remineralizing and demineralizing solutions against reference values (7.0 and 4.40, respectively), we found that only on day 3 in the demineralizing solution were no statistically significant differences observed (*p* = 0.139) (Table 2).

Table 3 presents the changes in pH in the demineralizing and remineralizing solutions between the control and experimental groups. The only significant difference was observed between the self-ligating brackets with and without electropolishing (*p* = 0.035); however, in general, the groups with the highest amount of metal in their composition (brackets) showed greater acidification of both solutions.

**Figure 5:**
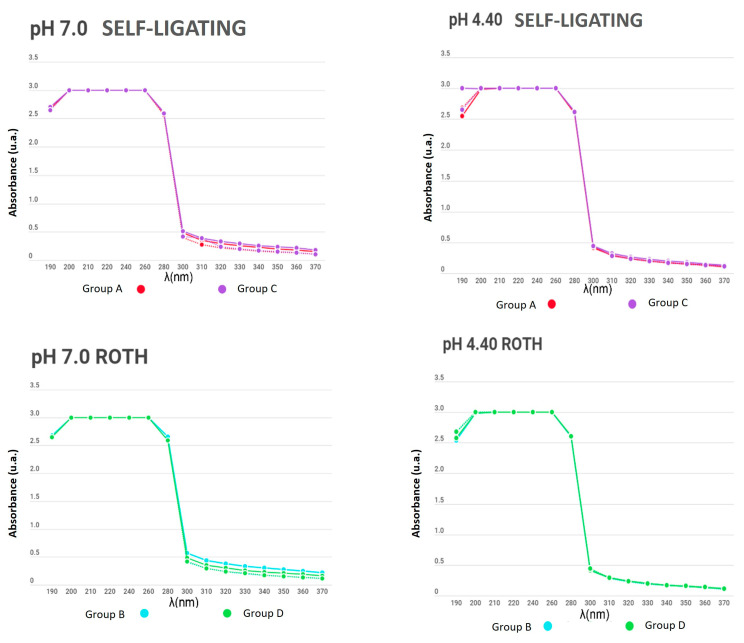
Absorbance between the remineralizing and demineralizing solutions in bracket groups (from 190 to 370 nm).

**Figure 6:**
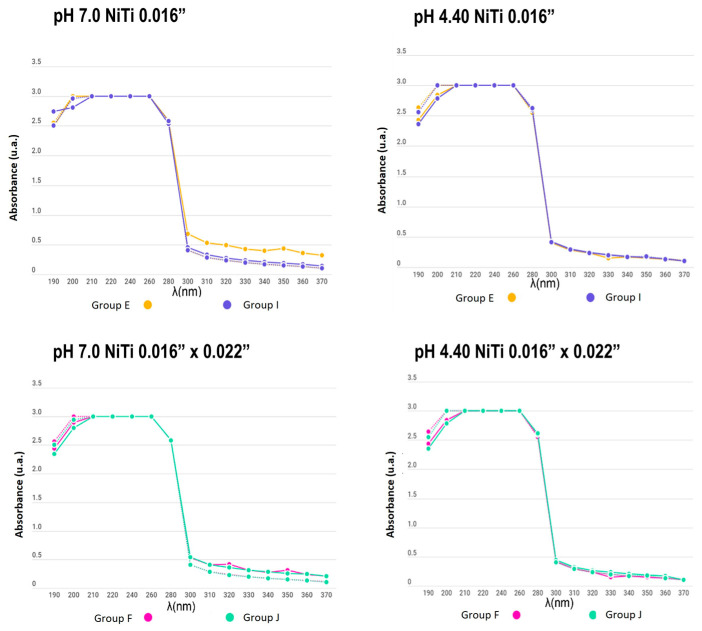
Absorbance between the remineralizing and demineralizing solutions in NiTi archwire groups (from 190 to 370 nm).

**Figure 7:**
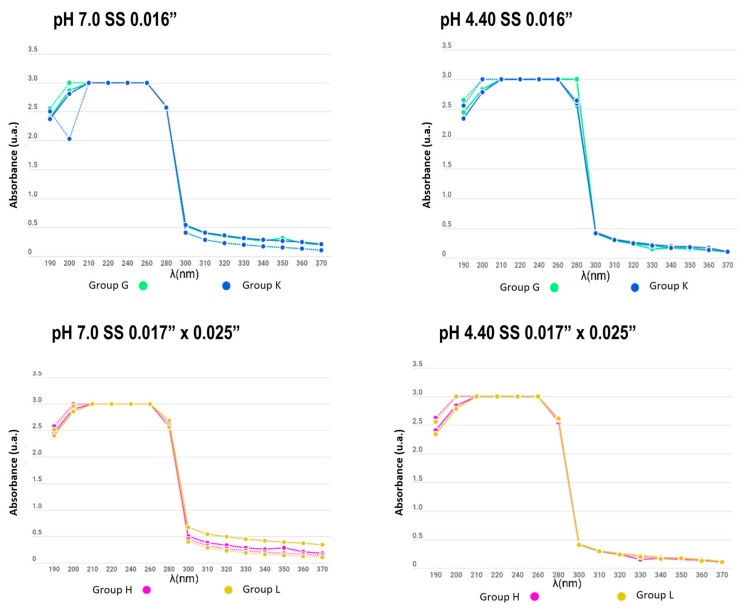
Absorbance between the remineralizing and demineralizing solutions in SS archwire groups (from 190 to 370 nm).

The absorbance between the remineralizing and demineralizing solutions did not differ significantly during the pH cycle (3, 5, 7, and 15 days after). Non-significant metallic ions were found in the solutions, and both the control and experimental groups maintained the initial conditions. In Figure 5, Figure 6 and Figure 7, it is observed that the solid lines refer to the behaviour of the solution on day 1 and the dotted line refers to the behaviour of the solution on day 15, which generally did not present differences in their behaviour.

### 3.2. SEM Characterization

**Figure 8:**
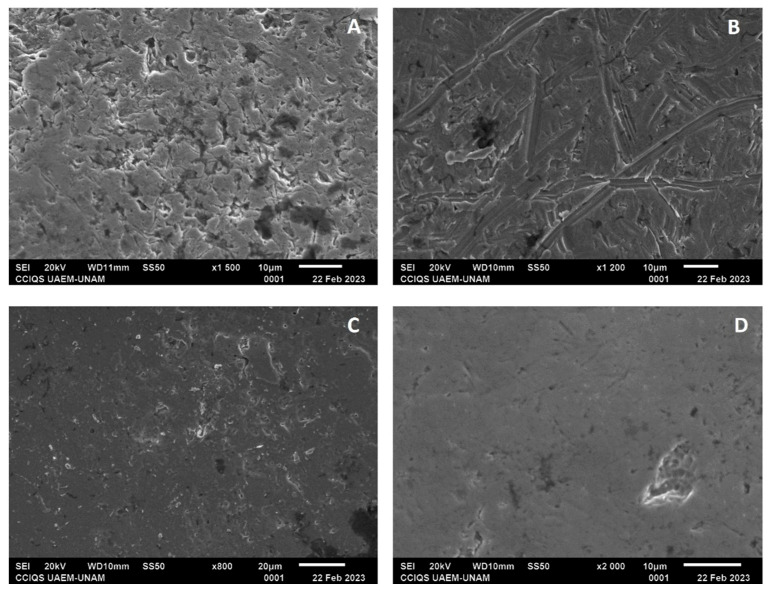
(**A**) Bracket from GA with a magnification of ×1500; (**B**) bracket from GC with a magnification of ×1200; (**C**) bracket from GB with a magnification of ×800; and (**D**) bracket from GD with a magnification of ×2000.

From the SEM characterization, the self-ligating brackets, GA, showed irregular surfaces with multiple pores, while in GC, after electropolishing, surfaces with smooth and continuous lines and with less deep irregularities were observed (Figure 8A,B). In the elemental analysis (EDSX analysis) of GA, the proportion of chromium was found to be 26.89%; that of iron, 73.56%; and that of nickel, 8.17%; for GC, the proportion of chromium was found to be 29.30%; that of iron, 61.18%; and that of nickel, 17.10%.

In the Roth technique groups, the same surface conditions as those of the self-ligating groups were observed for GB and GD. In GB, the proportion of chromium was found to be 24.21%; that of iron 4.15%; and that of cobalt, 67.49%. In contrast, for GD, the proportion of chromium was found to be 24.45%; that of iron, 4.10%; and that of cobalt, 67.88% (Figure 8C,D).

**Figure 9:**
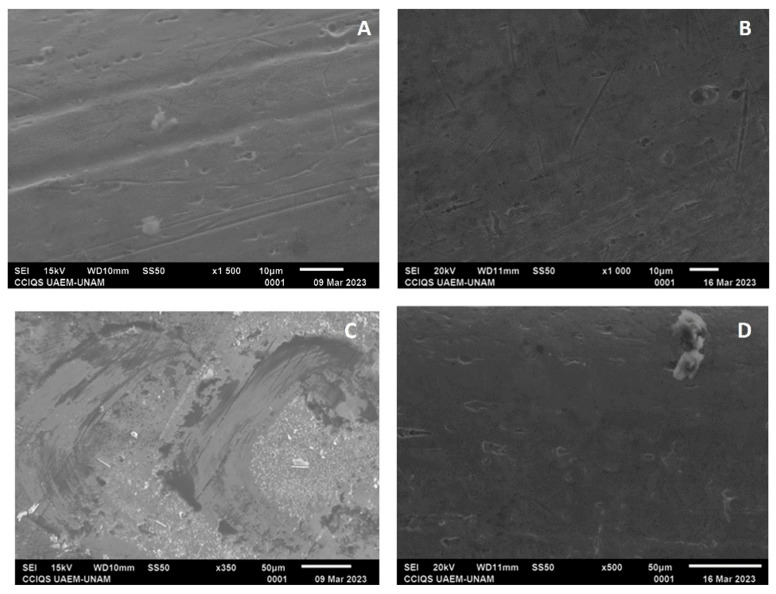
(**A**) Archwire from GE at ×1500 magnification; (**B**) archwire from GI with ×1000 magnification; (**C**) archwire from GF at ×350 magnification; and (**D**) archwire from GJ at ×500 magnification.

**Figure 10:**
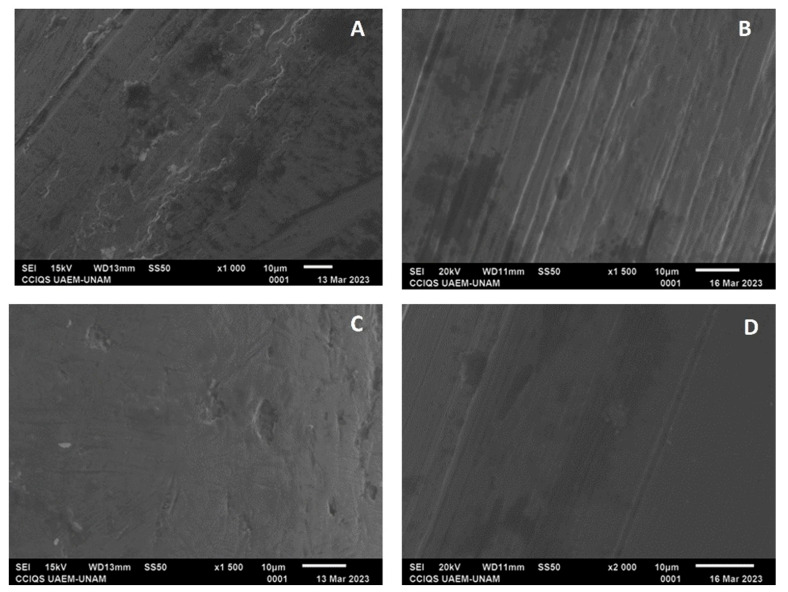
(**A**) Archwire from GG at ×1000 magnification; (**B**) archwire from GK at ×1500 magnification; (**C**) archwire from GH at ×1500 magnification; and (**D**) archwire from GL at ×2000 magnification.

For all the archwire groups, some irregularities with smooth lines were observed; however, in the experimental groups, in general, the surfaces were observed to have greater smoothness (Figure 9 and Figure 10).

Regarding the NiTi archwire groups, GE had a proportion of nickel at 18.97% and that of titanium at 32.54%, while GI had a proportion of nickel at 0.0% and that of titanium at 52.58%. For GF, the proportions were found to be 37.1% for nickel and 33.96% for titanium; meanwhile for GJ, nickel showed a proportion of 41.42% and titanium of 47.64%.

For stainless steel archwires, GG showed proportions of 3.38% nickel, 45.93% iron, 13.03% chromium, and 28.94% carbon; for GK, the proportions were 5.29% nickel, 56.34% iron, 15.61% chromium, and 34.12% carbon. Furthermore, GH consisted of 5.93% nickel, 53.81% iron, 15.05% chromium, and 22.25% carbon, while GL showed proportions of 7.28% nickel, 66.48% iron, 19.24% chromium, and 19.22% carbon.

## 4. Discussion

A previous study [11] showed that orthodontic appliances release nickel and titanium into the oral cavity, with increased levels in the saliva 3 months after the placement of the orthodontic metallic appliance. Metal ion concentrations in the urine also increased at 3 and 6 months after starting the orthodontic treatment. Therefore, finding their values in systemic secretions could give us clues about their circulation in the body. Furthermore, a SEM analysis showed that both stainless steel and NiTi archwires had signs of metallic corrosion.

However, electropolishing treatments on metal surfaces at an industrial level have been useful in improving such characteristics. Electropolishing has also been increasingly used to finish the surface of instruments in the medical field to facilitate the cleaning of surgical equipment and to maintain very low levels of contamination [14].

During the electrochemical polishing process, the surface is smoothed without using mechanical tools; thus, the surface layer is protected against structural changes. With this process, uniform passivation occurs on the surface of the polished material, which protects it against corrosion, and alongside hard access, gives it an aesthetic appearance and biocompatibility. There is a growing demand for electropolished titanium, aluminium, niobium, copper, and nickel materials for applications that include superconducting channels, and micro-electrochemical and bio-medical products [10,15].

Different pieces of metal finished with mechanical polishing or electropolishing were previously compared, with the latter showing better anti-corrosion properties. The surface layers of the austenitic metals treated with electropolishing turned out to be more resistant to pitting corrosion than those that were polished mechanically [16].

Regarding the electropolishing procedure, different electrolytic substances were evaluated at different temperatures, and the one with the best results was 35% sulfuric acid and 45% orthophosphoric acid, with an ideal temperature of 35 °C [17].

In terms of applications in the dental field, the use of electropolishing in endodontic instruments composed of NiTi has been reported to have benefits in prolonging fatigue life, as well as reducing surface irregularities, which are known to concentrate stress and initiate cracks. Other studies have also been carried out on resistance to torsion, fatigue, and cutting efficiency, showing that electropolishing treatments improve these characteristics [18].

Munjal [19] demonstrated, using SEM, that archwires subjected to electropolishing obtained polished and shiny surfaces; thus, they recommended the use of electropolishing at the office level. This procedure was carried out in a rudimentary way with a 0.005″ × 0.0180″ band used as the cathode in a glass container; the surface to be polished was submerged inside an electrolytic bath with tweezers, which acted as the anode. Then, it was connected to an electrical power supply emitting 12 V, and in this way, a complete electrical circuit was formed. In contrast, in our study, we used a special electropolishing tub with the temperature and voltage standardized, allowing for better control of these variables, and obtained shiny, smooth and irregular-free metallic surfaces.

A similar study was developed by Yoneyama and Hanawa [20], where they concluded that when NiTi wires receive electrolytic treatments, their resistance to corrosion improves and more biocompatible attachments form.

Fixed orthodontic appliances release nickel and chromium ions into saliva through electrochemical decomposition, which leads to the production of free radicals and potential chemical changes in the DNA base; although their values do not reach toxic levels, they can nonetheless cause hypersensitivity [21].

In the present study, the number of metal ions released into a solution was measured to compare appliances with and without electropolishing treatment; similar curves were obtained for all experimental and control samples, even for days with no significant change in the absorbance of the solutions. Additionally, no differences between groups were found due to the material of the used brackets and wires. The measurement of the substances was carried out for 15 days while subjecting the appliances to a pH cycle, so that the behaviour of the electropolished orthodontic appliances would mimic that when the appliance came directly from the manufacturer.

Orthodontic treatment has evolved over time, and fixed appliances commonly use brackets, including conventional brackets and self-ligating brackets. Self-ligating brackets are the most commonly utilized because clinicians find them comfortable to use. Their major advantage is that they have lower kinetic frictional forces than conventional brackets and do not use ligatures because they have a moveable component to entrap the archwire, avoiding a greater amount of bacteria; however, its formation requires a greater metal surface area [22].

When analysing the pH changes from the cyclic pH solutions, the group of self-ligating brackets showed the greatest changes, possibly due to their composition, as more than 60% of their surface structure was composed of iron, a material that corrodes easily, and the group with the least pH variation was GI, which may have been affected by the electropolishing increasing the proportion of titanium, as titanium is one of the most corrosion-resistant metals [23].

Regarding the characterization with SEM, the control groups showed greater irregularities with multiple pores compared with the experimental groups, which presented smoother surfaces, fewer lines, and fewer irregularities; in both groups, these irregularities may have been due to the pH cycle or may have come from their manufacture, despite the experimental groups showing changes in their surface compositions. The self-ligating bracket group showed a decrease in the amount of iron and an increase in the percentage of chromium; according to previous studies, electropolishing reduces the percentage of iron to leave a surface rich in chromium, and this phenomenon helps the passivation of the electropolished surface. The low ratio between iron oxide and total chromium oxides and hydroxides contributes to increasing the corrosion resistance of the passive layers obtained following electropolishing [24].

Concerning NiTi archwires, the amount of nickel decreased after electropolishing according to the EDSX analysis but the amount of titanium increased, which could reduce the risk of sensitivity or allergy to nickel, which is one of the considerations that must be taken when subjecting a patient to orthodontic treatment [21,24,25,26]; it could improve the corrosion resistance and facilitate the cleaning of attachments by eliminating bacteria retention areas [27].

The use of electropolishing on metals in dentistry is a recent development, so more research must be conducted to evaluate other properties, such as cytotoxicity, to measure the presence of metals in saliva or other biomarkers, decrease antibacterial adhesion, and determine physical properties, among others.

## 5. Conclusions

When determining the corrosion levels of orthodontic appliances with and without electropolishing, the number of metal ions released in a solution was observed to have a similar curve for all the experimental and control samples, even for the days with no significant change in the absorbance of the solutions.

The characterization of the surface of the experimental and control groups showed differences, with greater irregularities and multiple pores in the control groups compared to the experimental groups, which presented smoother surfaces, fewer lines, and fewer irregularities; the experimental groups showed a change in the surface composition, thus drawing attention to NiTi archwires and a decrease in nickel on their surfaces.

Therefore, electropolishing treatments in metallic orthodontic attachments do not affect their surface structure but, rather, possibly improve the shine, antibacterial adherence, and sliding in the orthodontic area; therefore, they are an option for patients with hypersensitivity to metals, especially nickel.

## Figures and Tables

**Table 1:** Average behaviour of cyclical pH solutions over time in the study groups.

Days	Groups											
	A	C	B	D	E	I	F	J	G	K	H	L
Remineralizing Solution												
1	6.10	6.76	6.87	6.87	6.87	6.94	6.75	6.70	6.84	6.64	6.87	6.76
3	6.12	6.45	6.56	6.11	6.54	6.10	6.11	6.40	6.10	6.45	6.34	6.54
7	5.95	6.10	6.15	5.93	6.25	5.96	6.03	5.81	6.06	6.01	6.13	6.09
15	5.07	5.66	5.62	6.28	6.54	6.41	6.53	6.18	6.52	6.74	6.51	6.20
Demineralizing Solution												
1	4.10	4.12	4.20	4.23	4.01	4.09	4.02	4.09	4.00	4.00	4.01	4.00
3	4.05	3.99	4.07	3.99	5.00	4.90	5.00	4.80	5.00	4.50	4.98	5.01
7	5.09	5.19	5.10	5.15	5.12	5.10	5.12	5.11	5.20	5.25	5.10	5.11
15	4.04	3.88	4.01	4.00	3.98	4.02	3.98	4.02	3.90	4.00	3.96	4.02

**Table 2:** Average pH values of solutions subjected to the pH cycle throughout the study against reference values.

	Remineralizing Solution		Demineralizing Solution	
Days	Mean ± SD	*p*	Mean ± SD	*p*
1	6.79 ± 0.09	0.000 *	4.07 ± 0.08	0.000 *
3	6.31 ± 0.19	0.000 *	4.60 ± 0.45	0.139
7	6.03 ± 0.11	0.000 *	5.13 ± 0.05	0.000 *
15	6.18 ± 0.49	0.000 *	3.98 ± 0.04	0.000 *

Student’s *t*-test for related samples * *p* < 0.05.

**Table 3:** Analysis of the pH solutions subjected to the pH cycle between the experimental and control groups.

	Remineralizing Solution		Demineralizing Solution	
Group	Mean ± SD	*p*	Mean ± SD	*p*
Brackets				
Experimental group—self-ligating (GA)	5.81 ± 0.49	0.035 *	4.32 ± 0.51	0.683
Control group—self-ligating (GC)	6.24 ± 0.47		4.29 ± 0.60	
Experimental group—Roth (GB)	6.30 ± 0.54	0.992	4.34 ± 0.50	0.936
Control group—Roth (GD)	6.29 ± 0.40		4.34 ± 0.54	
Archwires				
Experimental group—NiTi archwire 0.016” (GE)	6.55 ± 0.25	0.169	4.52 ± 0.61	1.000
Control group—NiTi archwire 0.016” (GI)	6.35 ± 0.43		4.52 ± 0.55	
Experimental group—NiTi archwire 0.016”× 0.022” (GF)	6.35 ± 0.34	0.593	4.53 ± 0.61	0.708
Control group—NiTi archwire 0.016”× 0.022” (GJ)	6.27 ± 0.37		4.50 ± 0.53	
Experimental group—SS archwire 0.016” (GG)	6.38 ± 0.37	0.568	4.52 ± 0.67	0.574
Control group—SS archwire 0.016” (K)	6.46 ± 0.32		4.43 ± 0.59	
Experimental group—SS archwire.017”× 0.025” (GH)	6.46 ± 0.31	0.581	4.51 ± 0.61	0.229
Control group—SS 0.017”× 0.025” (GL)	6.39 ± 0.30		4.53 ± 0.60	

SS = stainless steel; NiTi = nickel–titanium. Student’s *t*-test for related samples * *p* < 0.05.

## Data Availability

The data presented in this study are available upon request from the corresponding author.

## References

[1] Mikulewicz M., Wołowiec P., Michalak I., Chojnacka K., Czopor W., Berniczei-Royko A., Vegh A. (2014). Mapping chemical elements on the surface of orthodontic appliance by SEM-EDX. Med. Sci. Monit..

[2] Karnam S.K., Reddy A.N., Manjith C.M. (2012). Comparison of metal ion release from different bracket archwire combinations: An in vitro study. J. Contemp. Dent. Pract..

[3] Kusey R.P. (2002). Orthodontic biomaterials: From the past to the present. Angle Orthod..

[4] Gürsoy S., Acar A.G., Seşen C. (2005). Comparison of metal release from new and recycled bracket–archwire combinations. Angle Orthod..

[5] Maijer R., Smith D.C. (1982). Corrosion of orthodontic bracket bases. Am. J. Orthod. Dentofacial Orthop..

[6] Maijer R., Smith D.C. (1986). Biodegradation of orthodontic bracket system. Am. J. Orthod. Dentofacial Orthop..

[7] Huang H.H., Chiu Y.H., Lee T.H., Wu S.C., Yang H.W., Su K.H., Hsu C.-C. (2003). Ion release from NiTi orthodontic wires in artificial saliva with various acidities. Biomaterials.

[8] Peltonen L. (1979). Nickel sensitivity in the general population. Contact Dermatitis.

[9] Kim J., Park J.K., Kim H.K., Unnithan A.R., Kim C.S., Park C.H. (2017). Optimization of Electropolishing on NiTi Alloy Stents and Its Influence on Corrosion Behavior. J. Nanosci. Nanotechnol..

[10] Łyczkowska-Widłak E., Lochyński P., Nawrat G. (2020). Electrochemical Polishing of Austenitic Stainless Steels. Materials.

[11] Velasco-Ibáñez R., Lara-Carrillo E., Morales-Luckie R.A., Romero-Guzmán E.T., Toral-Rizo V.H., Ramírez-Cardona M., García-Hernández V., Medina-Solís C.E. (2020). Evaluation of the release of nickel and titanium under orthodontic treatment. Sci. Rep..

[12] Carrillo-Novia I., Lara-Carrillo E., Torres-Bugarin O., Morales-Valenzuela A.A., Salmerón-Valdés E.N., Hegazy-Hassan W., Velázquez-Enríquez U., Toral-Rizo V.H. (2023). Use of liquid-based cytology samples reveals genomic instability and cell death in patients undergoing orthodontic treatment. J. Oral Sci..

[13] Murdoch-Kinch C.A., McLean M.E. (2003). Minimally invasive dentistry. J. Am. Dent. Assoc..

[14] Han W., Fang F. (2020). Investigation of electrochemical properties of electropolishing Co–Cr dental alloy. J. Appl. Electrochem..

[15] Zaki S., Zhang N., Gilchrist M.D. (2022). Electropolishing and Shaping of Micro-Scale Metallic Features. Micromachines.

[16] Rokosz K., Solecki G., Mori G., Fluch R., Kapp M., Lahtinen J. (2020). Effect of polishing on electrochemical behavior and passive layer composition of different stainless steels. Materials.

[17] Núñez P.J., García-Plaza E., Hernando M., Trujillo R. (2013). Characterization of surface finish of electropolished stainless steel AISI 316L with varying electrolyte concentrations. Procedia Eng..

[18] Mohammadi Z., Soltani Mk Shalavi S., Asgary S. (2014). A Review of the various surface treatments of Niti instruments. Iran Endod..

[19] Munjal S., Duggal R., Singh N.A., Kaur A. (2014). Electropolishing -Orthodontic Office: A simplified approach. Clin. Innov..

[20] Yoneyama T., Hanawa T. (2020). Reduction in nickel content of the surface oxide layer on Ni-Ti alloy by electrolytic treatment. J. Oral Sci..

[21] Urbutyte K., Barciute A., Lopatiene K. (2023). The changes in nickel and chromium ion levels in saliva with fixed orthodontic appliances: A systematic review. Appl. Sci..

[22] Tiwari A., Aafaque S., Rizwana Y., Quadri S., Kanagasabapathy B., Villuri C., Babu J., Swarnalatha C. (2023). Canine retraction and anchorage loss using self-ligating and conventional brackets with sliding mechanics: A split-mouth clinical study. J. Orthod. Sci..

[23] Prando D., Brenna A., Diamanti M.V., Beretta S., Bolzoni F., Ormellese M., Pedeferri M. (2018). Corrosion of titanium: Part 2: Effects of surface treatments. J. Appl. Biomater. Funct. Mater..

[24] Haïdopoulos M., Turgeon S., Sarra-Bournet C., Laroche G., Mantovani D. (2006). Development of an optimized electrochemical process for subsequent coating of 316 stainless steel for stent applications. J. Mater. Sci. Mater. Electron..

[25] Pazzini C.A., Pereira L.J., Peconick A.P., Marques L.S., Paiva S.M. (2016). Nickel allergy: Blood and periodontal evaluation after orthodontic treatment. Acta Odontol. Latinoam..

[26] Markovic E., Peric T., Kojic S., Stosic M., Scepan I., Petrovic B. (2024). Influence of casein phosphopeptide-amorphous calcium phosphate on the surface topography and composition of nickel-titanium archwires during orthodontic treatment with fixed appliances. J. Oral Sci..

[27] Thiruvengadam V., Chitharanjan A.B., Kumar K., Singaram V. (2022). Comparison of Streptococcus mutans Adhesion on New and Recycled Metal Brackets: An in Vitro Study. Cureus.

